# Nanobodies that Neutralize HIV

**DOI:** 10.3390/vaccines7030077

**Published:** 2019-07-31

**Authors:** Robin A. Weiss, C. Theo Verrips

**Affiliations:** 1 Division of Infection & Immunity, University College London, 90 Gower Street, London WC1E 6BT, UK; 2 QVQ Holding bv, Padualaan 8, 3584 CL Utrecht, The Netherlands

**Keywords:** nanobody, VHH, neutralization, HIV-1, vaccine design, microbicide

## Abstract

Nanobodies or VHH (variable domains of heavy-chain only antibodies) are derived from camelid species such as llamas and camels. Nanobodies isolated and selected through phage display can neutralize a broad range of human immunodeficiency virus type 1 (HIV-1) strains. Nanobodies fit into canyons on the HIV envelope that may not be accessible to IgG (immunoglobulin G) containing both heavy and light chains, and they tend to have long CDR3 (complementarity-determining region 3) loops that further enhance recognition of otherwise cryptic epitopes. Nanobodies are readily expressed at high levels in bacteria and yeast, as well as by viral vectors, and they form relatively stable, heat-resistant molecules. Nanobodies can be linked to human Fc chains to gain immune effector functions. Bivalent and trivalent nanobodies recognizing the same or distinct epitopes on the envelope glycoproteins, gp120 and gp41, greatly increase the potency of HIV-1 neutralization. Nanobodies have potential applications for HIV-1 diagnostics, vaccine design, microbicides, immunoprophylaxis, and immunotherapy.

## 1. Introduction

**Figure 1:**
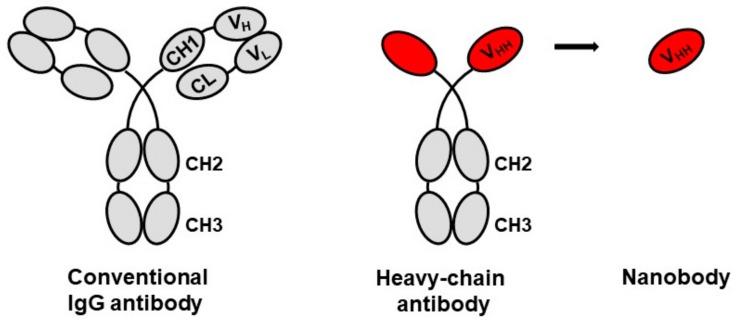
Schematic of conventional immunoglobulin (IgG) antibody and camelid heavy-chain only antibody with derivation of a nanobody representing the N-terminal variable antigen recognition region.

Nanobodies represent the Variable regions of the Heavy chain of Heavy-chain only (VHH) immunoglobulins (Figure 1). Heavy-chain only antibodies occur naturally in members of the *Camelidae*, comprising Old World species, camels and dromedaries, and New World species, such as llamas, alpacas, and vicuñas [1]. The occurrence of functional antibodies lacking light chains in camels, and the potential for exploiting the variable region (VHH) without needing a matching light chain, was first documented in 1993 [2]. Camelids also make conventional immunoglobulin G (IgG), but the heavy chain only antibodies are encoded by distinct germ-line genes from IgG and represent approximately 30% of circulating antibodies. Both sets of antibodies respond to immunogens by somatic rearrangement and mutation resulting in affinity maturation to synthesize high affinity specific antibodies. Cartilaginous fish (Elasmobranchs) such as sharks also make heavy chain only antibodies [3,4]. It is not known why both camelids and sharks express both kinds of antibody, but the persistence of single chain antibodies among them indicates a selective advantage to maintaining them [4]. The designation Nanobody® for camelid single chain variable regions was originally claimed as a brand name for what is more accurately called VHH, but the term nanobody has come into general usage interchangeably with VHH. Perhaps ‘minibody’ would be a more accurate term, since nanobodies comprise nearly 10% of the molecular weight of a conventional IgG with complete heavy chains and light chains.

Since nanobodies are small proteins of ~12–15 kDa, yet possess the binding specificity and affinity of antibodies, they have many uses in biotechnology and medicine [1]. They help to stiffen flexible proteins to aid crystallization and structural studies [1,5] and block enzyme function [1], and are used for tracking intracellular and cell surface proteins in cell biology [6,7]. As examples pertinent to HIV research, nanobodies 238D2 and 238D4 bind to cellular CXCR4 (C-X-C chemokine receptor type 4 or CD184) and neutralize X4 strains of HIV-1 by blocking this coreceptor [8], intracellular nanobodies (intrabodies) to Rev inhibit multimerization to interfere with virus production [9], and an anti-p24 nanobody has been employed as a detector of p24 Gag antigen in designing miniaturized diagnostic tests of HIV-1 infection [10,11].

Virus-neutralizing nanobodies have been developed to several animal and human virus families in addition to HIV-1. These include rotaviruses [12], influenza A viruses [13,14], respiratory syncytial virus [13], norovirus [15], and rabies virus [13]. In murine models, nanobodies prevent disease in H5N1 influenza [16] and neonatal rotavirus infection [17]. Alternative small protein fragments to VHH with specific ligand properties have also been investigated for neutralization of HIV-1 and for exploitation as potential microbicides or therapeutics. They include, for example, the outer domains of the CD4 (cluster of differentiation antigen 4) receptor linked to an IgG framework [18] or expressed in an adeno-associated virus (AAV) vector [19], and designed ankyrin repeat proteins (‘Darpins’) [20,21]. In this review we focus on the properties and potential applications of HIV-1-neutralizing nanobodies.

## 2. HIV-1 Broadly Neutralizing Nanobodies

### 2.1. Isolation and Characeterization of HIV-1 Neutralizing Nanobodies IUV-1, for Instance

Over the past 10 years, several highly potent and broadly neutralizing monoclonal antibodies (bnmAbs) have been derived from ‘elite neutralizer’ patients who suppress their HIV-1 load partly through humoral immunity [22,23]. Several of these human bnmAbs recognize conformational epitopes that are highly dependent on the native conformation of gp120 and gp41 including glycan residues on the HIV-1 envelope. In contrast, antisera and mAbs from experimental immunization of animals seldom exhibit high titer genuinely broad and conformation-dependent properties [23,24], despite the worldwide effort to develop HIV vaccines.

Table 1 lists the HIV-1-neutralizing camelid nanobodies of which we are aware [25,26,27,28,29,30,31,32,33]. Broadly neutralizing nanobodies were isolated from llamas *(Lama glama)* [25,26,27,28,29,30,31] or dromedaries (*Camelus dromedarius*) [32], which were immunized with recombinant gp120 or gp140, and in one study with proteoliposomes incorporating gp41 [28]. The llama nanobodies reported to date, however, do not map to the sites most sensitive to tertiary or quaternary conformation, probably because the recombinant antigens used for immunization were partly unfolded for these sites and therefore represent suboptimal immunogens.

Although a ‘SOSIP’ (a gp140 genetically engineered by insertion of cysteine residues to create a stable disulfide bond between gp120 and gp41) was used most recently as the immunogen [32], the VHH recognized the CD4bs, and it will be interesting to learn whether nanobodies to conformation-dependent epitopes can also be identified in this VHH library.

**Figure 2:**
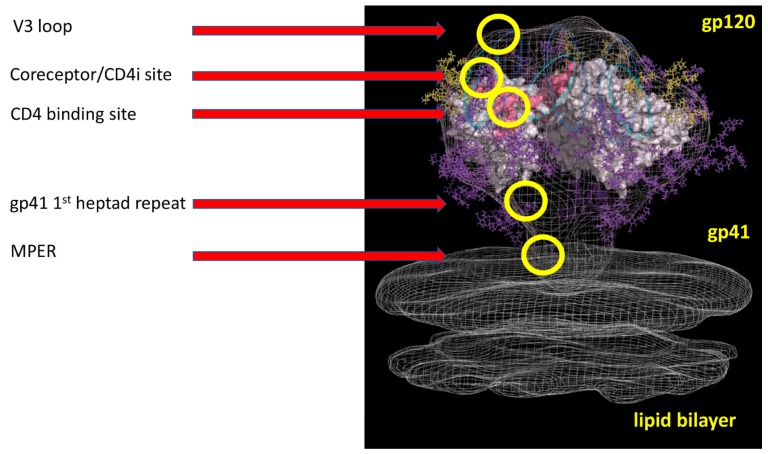
Schematic diagram of the HIV-1 trimeric envelope glycoprotein complex showing the approximate locations of domains in yellow circles that are recognized by the broadly neutralizing nanobodies listed in Table 1.

As shown in Figure 2, the nanobodies recognize five domains of the HIV-1 envelope, three epitopes on gp120 and two on gp41. The target sites are (a) the CD4 binding site (CD4bs), (b) a CD4-induced domain overlapping the chemokine coreceptor site and CD4bs, (c) the crown of the V3 loop, (d) the first heptad repeat on gp41 of the fusion domain, and (e) the membrane proximal envelope region (MPER) on gp41. Four of these domains were already well known from studies of human antibodies, while the linear epitope on the first heptad repeat of gp41 [33] is close to the epitope of human mAbs D5 and HK20 [34] a site which is transiently exposed in the fusion-intermediate conformation. To date, then, experimentally derived nanobodies have not revealed totally novel neutralization sites although they provide useful tools for analysis and application, as described later.

In the initial investigation of nanobodies that neutralize HIV-1, llamas were immunized with monomeric gp120 and phage libraries of cDNA of VHH were prepared [25]. To seek nanobodies to the CD4bs, VHH were first selected by panning on gp120, and then those that recognize the CD4bs were selectively eluted by high concentrations of soluble CD4 antigen. Three VHH, A12, C8, and D17, were identified as having ‘broad’ neutralization properties by the standards of the time in covering 40–50% strains tested, equivalent to the broadest known human mAb in 2008, b12. This study provided proof of principal that HIV-neutralizing single chain antibodies can be elicited in camelids by experimental immunization [25].

**Figure 3:**
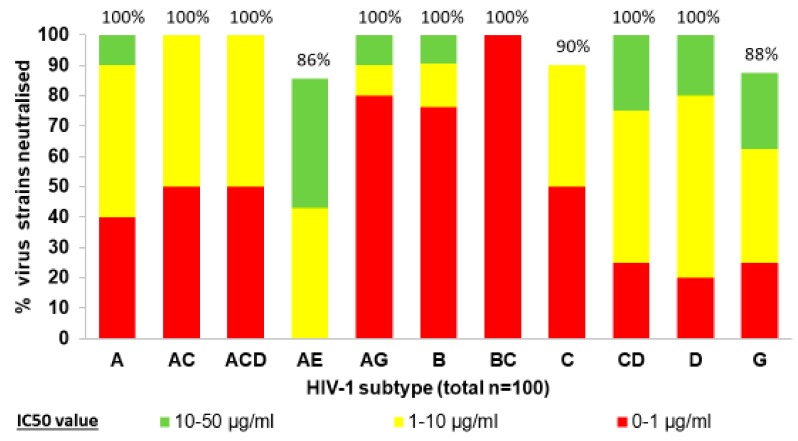
Potency of neutralization by CD4bs nanobody J3. Ninety-six of 100 HIV-1 pseudoviruses of various subtypes and circulating recombinant strains were sensitive to neutralization. Reproduced with permission from McCoy et al. [27].

Anti-gp41 nanobodies were isolated from llamas immunized either with gp140 [26], or with proteoliposomes bearing gp41 [28]. Further anti-gp120 nanobodies were isolated from llamas immunized with recombinant gp140 by primary screening for neutralization and resulted in two further nanobodies from different llamas, J3 [27] and 3E3 [26,33], which each exhibit a high affinity to the CD4bs titer and a breadth of neutralization of >95% of strains, as shown for J3 in Figure 3. Pseudoviruses constructed from multiple HIV-1 Group M subtypes and circulating recombinant strains were tested, including those classified as comprising more resistant tier 2 and tier 3 envelopes [35]. Nanobodies J3 and 3E3 showed a similar potency and breadth as the human CD4bs mAb, VRC01, and a combination neutralized 99% of the strains tested [33]. Anti-CD4bs nanobodies also neutralized simian-human hybrid SHIV viruses [27,31] but only weakly affected Simian Immunodeficiency Virus (SIV) isolated from chimpanzees and gorillas [36]. Matz et al. obtained an interesting set of nanobodies recognizing a CD4-induced epitope that includes elements of coreceptor and CD4 binding sites [29,30]. Thus, for certain epitopes, experimental immunization of llamas even with ‘sub-prime’ immunogens yielded nanobodies as potent as those from human elite controllers [23].

### 2.2. Structural Studies of HIV-1 Nanobodies

Structural studies of the interaction of human monoclonal antibodies with the HIV envelope have been most helpful in defining epitopes and in indicating possible routes for improving vaccine design. For example, the CD4 binding ‘supersite’ on gp120 has been subject to detailed analysis by co-crystallization of gp120 and several human mAbs [37]. Nanobodies are usually amenable to crystallization in unliganded form and they also help to stabilize larger proteins for structural studies [5,38]. Peter Kwong’s group solved the crystal structure of VHH JM4 [30] bound to gp120; they have also resolved to high resolution VHH J3 bound to gp120, which shows similar binding and angle of approach to the CD4 binding pocket as soluble CD4 itself (C. Theo Verrips, personal communication).

**Figure 4:**
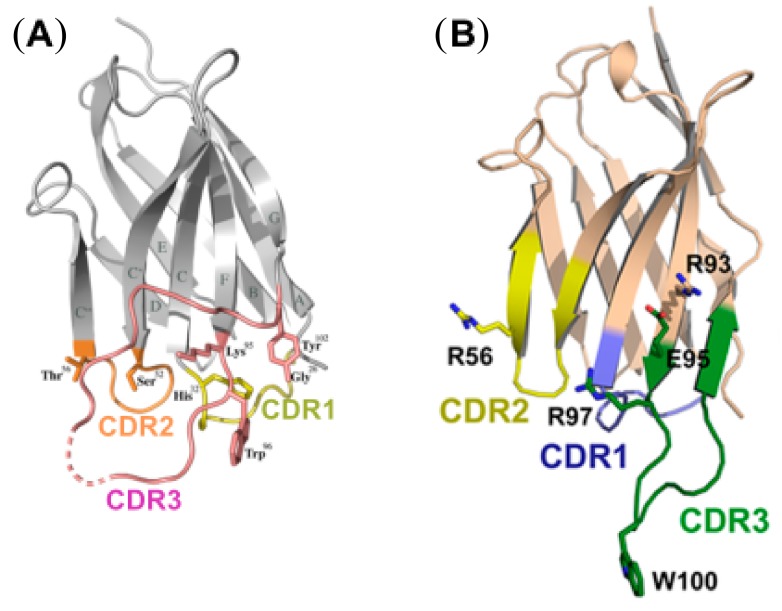
Crystal structures of anti-HIV-1 nanobodies. (**A**) Ribbon representation of CD4bs VHH D7; the complementarity determining regions (CDR) are highlighted in yellow (CDR1), orange (CDR2), and pink (CDR3). The side chain of tryptophan Trp96 on CDR3 is critical for gp120 interaction and neutralization. The dotted line indicates CDR3 residues without sufficient resolution. Reproduced with permission from Hinz et al. [39]. (**B**) Ribbon representation of membrane proximal envelope region (MPER) region nanobody 2H10. The CDR loops are colored blue (CDR1), yellow (CDR2), and green (CDR3). The hydrophobic tryptophan residue at the tip of the long CDR3 loop is not required for binding to gp41 but is essential for orientation in the lipid envelope of the virus and for weak neutralization activity; much stronger neutralization resulted by ligating two 2H10 VHH via a glycine-serine linker. Reproduced with permission from Lutje Hulsik et al. [28].

Figure 4 shows the structure of the D7 CD4bs nanobody and the anti-gp41 MPER nanobody, 2H10, analyzed by Winfried Weissenhorn’s group [28,39]. It is noteworthy that llama nanobodies typically have a long CDR3 loop, as subsequently shown with other ruminant species that produce HIV-neutralizing conventional antibodies [40]. The MPER nanobody 2H10 (Figure 4B) bears a tryptophan residue at the tip of the CDR3 loop which is thought to interact with the lipid bilayer. Mutational analysis demonstrated that while this hydrophobic residue did not alter the binding property of 2H10 to gp41, it is crucial for HIV-1 neutralization [28].

**Figure 5:**
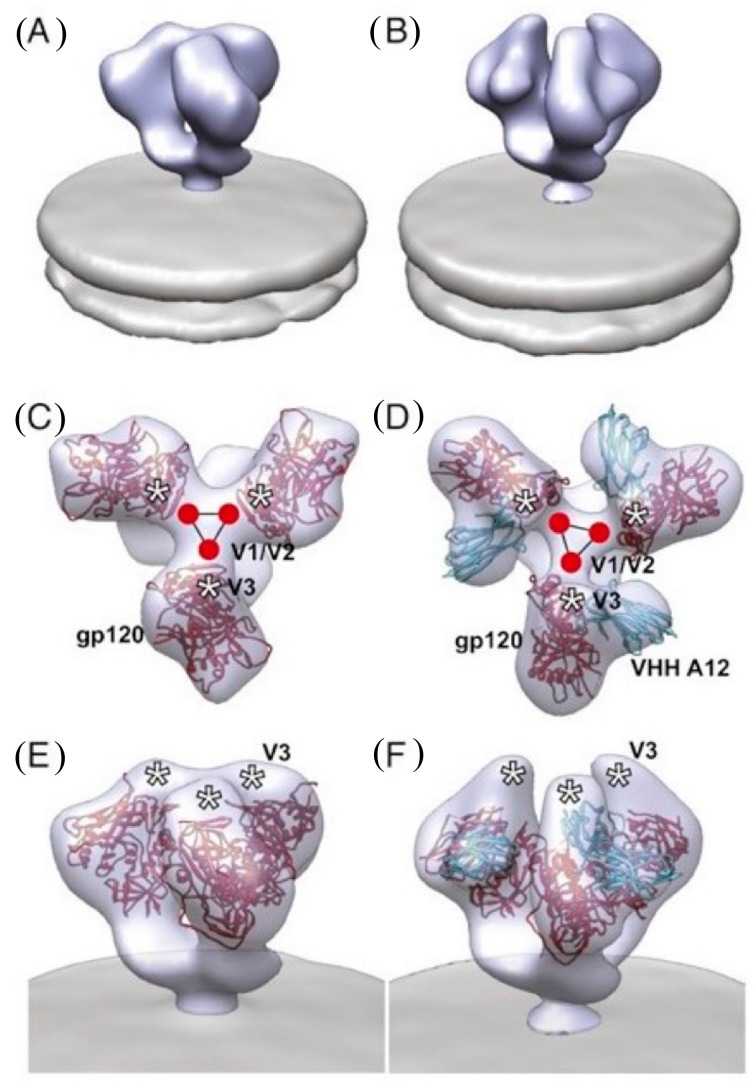
Cryo-electron microscopic tomograms of gp160 trimers on HIV particles with and without bound complementarity-determining (CD)4bs nanobody A12. (**A**,**B**): Perspective views of density maps for Envelope Trimer (Env) in the unbound (**A**) and A12-bound (**B**) states. (**C**,**D**): Top view and (**E**,**F**): Perspective view of the density map and molecular architecture of unbound and A12-bound trimeric Env, respectively. Cocrystal coordinate models show gp120 (red) and variable domains of heavy-chain only antibodies (VHH) A12 (cyan). The estimated locations of the V1/V2 and V3 loops are highlighted by the red circles and white asterisks, respectively. The partially open conformation of the V3 loop region in the A12-bound form is evident in (**F**). Reproduced by permission from Meyerson et al. [41].

Electron microscopic analysis of gp160 spikes on virus particles also aids the 3-dimensional modelling by tomography of the HIV-1 envelope trimer and the interaction of mAbs with it. Figure 5 depicts a cryoelectron tomogram of the trimeric envelope on HIV virions with and without the CD4bs nanobody A12 studied by Subramaniam’s group [41]. The small size of the nanobody nicely illustrates the structure of the trimer and its partially open structure induced after binding of the ligand to the CD4bs.

### 2.3. Molecular Manipulation and Modification of Anti-HIV-1 Nanobodies

#### Selection and Induction of Nanobody Variants

Once a nanobody has been identified, its specific sequence can be used to design primers to amplify other members of the same family from the phage library. This was performed with the CD4bs nanobodies A12 and D7 [25] which belong to a single maturation family [42]. While it was hoped that this approach would lead to the identification of related nanobodies with more potency or more breadth, A12 and D7 proved to be among the best in the family. Experimental mutatagenesis of the VHH genes, however, led to variants with subtly different properties and increased breadth of neutralization [42].

It later became possible to employ next generation deep sequencing to examine the molecular evolution of anti-HIV-1 heavy-chain antibody genes following immunization with gp140. The previously immunized llamas that gave rise to J3 and 3E3 CD4bs nanobodies were investigated together with two further llamas from which three broadly neutralizing nanobodies were identified [31]. It was clear that the nanobodies were matured fully only in response to the protein immunogens. The VHH elicited in different animals, while sharing functional hallmarks, were encoded by distinct sequences and thus could not have been identified by a deep sequencing analysis alone. By using ‘difficult-to-neutralize’ tier 2 and tier 3 HIV-1 strains for screening [35], it was possible to detect increased breadth of neutralization. Moreover, the potency and breadth could be improved by additional affinity maturation in vivo, or by artificially inducing mutations in vitro of residues predicted to provide higher affinity interaction with the viral envelope [31].

It is also possible to link a nanobody to other molecules such as Fc domains in order to reconstruct functional antibody molecules [16,43]. When linked to human Fc as described below for humanization, the nanobody gains bivalency, effector functions, and efficacy in blocking cell–cell spread of HIV-1 [43]. VHH JM4 also showed increased potency for preventing cell–cell spread when tethered to cell membranes by a C-terminal glycosylphosphatidylinositol-anchored (gpi) anchor [44], presumably because the VHH would be anchored at the site of virus spread known as the virological synapse [43].

### 2.4. ‘Humanized’ Reconstituted Antibodies with Nanobody Heads

Newly selected sequences of nanobodies differ in 10–14 amino acids from the most closely related human V-gene encoding the first 3 Frame Works [FW] and CDR1 and 2. These differences may result in undesirable immune responses when the nanobody is used in the human body. Vincke et al. have compared the sequences of camelid and human antibodies [45]. Llamas have 23 V-genes (unpublished results) and 7 J-genes. The 23 V-genes can be divided into 3 groups, mainly on basis of the fundamental difference of the amino acids 43–47 located in FW 2 [45,46]. Moreover, DeSchacht et al. found that VHH can be formed from conventional V3-genes [47]. By determining the likely parental gene sequences for the four classes of camelid single heavy chain Ig genes, each newly selected nanobody can be compared with the most closely related V- and J genes of humans [46].

Since there is now detailed information on the structure of conventional and camelid antibodies, the consequences of ‘humanizing’ framework amino acids can also be determined in silico. The company Ablynx bv (www.ablynx.com) has developed many humanized nanobodies, several of which are in clinical trials. Careful comparison of the differences between the parental nanobody and human sequences indicate that such nanobodies intended for potential clinical use are >90% humanized, which means that about half of the amino acids in the parental nanobody has been changed to those found in the human framework of heavy chain variable domains.

Another step towards humanization is to couple human Fc to humanized VHH. It is possible to attach VHH to human Fc regions of the human heavy chain and gain a hybrid antibody with effector functions such Fc receptor interaction and sensitivity to complement [43]. Notably, while J3 displayed a high level of potency in blocking cell-to-cell infection, the small size of the nanobody is not required for efficient neutralization of contact spreading infection since recombinant J3 containing a full-length human heavy chain Fc domain was significantly more potent. The increased neutralization potency of the reconstituted J3-Fc hybrid antibody may be due to its bi-valent structure because it would therefore possess greater avidity for the envelope [43].

### 2.5. Mono- and Bispecific Bi-Head and Tri-Head Nanobodies

It is possible to join two or three nanobodies together with glycine-serine linkers of various lengths. When this was done with anti-HIV nanobodies, enhanced neutralization was evident [28,29,33]. Homo bi-heads will have greater avidity than nanobodies with only one attachment site but must be able to reach across to the epitope on an adjacent member of the envelope trimer. This appears possible where the two epitopes are close together, such as the narrow MPER region of gp41. While the mono-head VHH 2H10 exhibited barely detectable neutralization, the bi-head was strongly neutralizing [28]. Matz et al. [29] constructed bivalent and trivalent nanobodies, such as VHH JM3 × 3, which extended the breadth of HIV-1 neutralization.

Conventional antibodies such as human mAbs have been constructed with heterologous variable regions which have shown increased efficacy of HIV-1 neutralization [48]. Mouquet et al. [49] constructed single chain V_H_ and V_L_ chains linked to the Fc region with heterodimers between epitopes on gp120 and gp41 which yielded powerful neutralizing Abs. Asokan et al. [50] found increased breadth and potency of some combinations, of which a bi-specific single chain antibody combining CD4bs VRC07 and PG16 mAbs was particularly effective. Taking a cue from heterologous human mAbs, bivalent HIV nanobodies with heterologous heads have a place in vaccine and therapeutic research [29,33]. Some bispecific bi-heads can freeze the virus envelope in a non-functional state by binding to two different sites presumably situated on the same glycoprotein of the trimer. The most potent bi-head combination we discerned was between the CD4bs on gp120 and the first heptad repeat on gp41 which increased neutralization potency 1400-fold [33].

### 2.6. Intracellular Expression of Nanobodies to Combat HIV

Like conventional antibodies, camelid heavy chain antibodies attain their tertiary, 3-dimensional conformation after folding in the endoplasmic reticulum (ER), and only correctly folded antibodies will be secreted. However, Dolk et al. [51] showed that after denaturation, a VHH refolded almost perfectly even in the absence of the redox enzymes, provided that the cognate antigen is present. Using this knowledge, several groups isolated nanobodies against the intracellular HIV-1 proteins, Rev, Nef and Vpr, which are crucial for the replication of HIV-1 in human cells. Vercruyse et al. selected nanobodies which interfere with the assembly of the Rev multimeric protein complex for the processing and export of viral RNA [9]. Bouchet et al. showed that intracellular expression of a nanobody specific to Nef blocks the HIV-1 replication cycle [52]. Matz et al. elegantly exploited a yeast two hybrid approach to select a single domain antibody that binds to Vpr and prevents its translocation from the cytoplasm to the nucleus [53]. Unfortunately, it has not yet become feasible to transport intact VHH into the cytoplasm of mammalian cells for unknown reasons [54].

## 3. Application of Nanobodies for Clinical Use Including Prevention and Treatment of HIV Infection

### 3.1. Production of Nanobodies

Despite considerable improvement of the yield of production of conventional antibodies, for low cost products like microbicides or veterinary imaging agents, the production costs remain too high. Fortunately, the production of VHH in bacteria and yeast is usually excellent. Whereas *E. coli* is mainly used in research laboratories as the production system for small amounts of VHH, the yeast *S. cerevisiae* is best suited to larger scale production [55]. A considerable advantage of yeast production is that the nanobody can usually be purified in one or two steps and will be free of endotoxin, which is a requirement for use in humans and animals.

### 3.2. Nanobody Expression in Commensal Lactobacillus Strains

A promising production and delivery system for nanobodies is to exploit lactic acid bacteria, which are common inhabitants of the gastro-intestinal (GI) [56] and genital tracts. Different *Lactobacillus* species inhabit various regions of the GI tract and the vagina which can be adapted as vectors for nanobodies. The group of Hammarström and Marcotte have developed various lactic acid bacteria to produce nanobodies in the GI tract. *L*. *paracasei* expressed nanobodies that neutralize the effect of toxin B of *Clostridium difficile* and reduced its virulence in a hamster model [57]. They showed that nanobodies neutralizing rotavirus are effective in *Lactobacillus* vectors in vivo, including bispecific ones [58]. Commensal vaginal *Lactobacillus* strains could also be adapted as nanobody expression vectors [59], including those prevalent in Africa [60]. Thus, *Lactobacilli* with a long residence period in the vagina can be developed as an in vivo delivery system for antibodies including nanobodies.

Lagenaur and colleagues demonstrated that when *Lactobacillus jensenii* was genetically engineered to express the HIV-1 inhibitor cyanovirin-N, it reduced the incidence of SHIV infection upon vaginal challenge in rhesus macaques [61], providing proof of principle that *Lactobacilli* can deliver anti-HIV-1 agents. Moreover, her group went on to show excellent antibody production by *L*. *jensenii* of a single chain antibody fragment, dAb-m36, derived from a broadly neutralizing human mAb recognizing the CD4-induced coreceptor site [62]. H. Marcotte and colleagues (manuscript in preparation) have produced both secreted and bacterium-tethered forms of VHH A12 and J3 that maintain excellent HIV-1-neutralizing properties. It currently remains an open question whether lactic acid bacteria are the optimal strategy compared to direct administration of nanobodies to food products to combat pathogens in the GI tract, or to gels for the control of pathogens in the vagina, but the potential of *Lactobacillus* vectors for prolonged VHH production is evident [56].

### 3.3. Nanobodies as Potential HIV Microbicides

**Figure 6:**
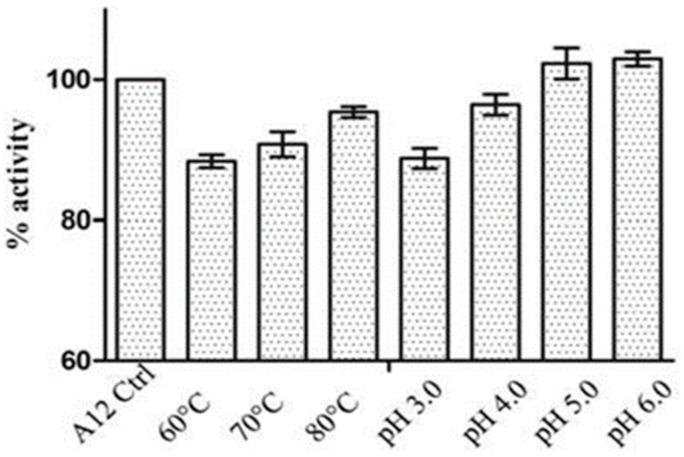
Resistance of CD4bs nanobody A12 to heat inactivation and to changes in pH shown as % of untreated neutralizing activity. It is not clear why the higher temperature of 80 °C retained greater activity than 60 °C and 70 °C; while statistically significant (bars depict standard errors), it probably represents variation between experimental readings. Reproduced with permission from Gorlani et al. [64].

Nanobodies are suitable candidates for development as vaginal microbicides and for reducing viral load after infection. We have noted above that nanobodies are able to refold after denaturation especially when bound to their antigen [51]. Gorlani et al. [63] demonstrated that an HIV-neutralizing CD4bs nanobody, A12, is resistant to denaturation up to 80 °C (Figure 6) which means that they will not need a cold chain for delivery in tropical climates. VHH A12 is also resilient against denaturation across a large range of pH values (Figure 6). This broad pH stability at slightly acid conditions is useful because, while the normal pH in the vagina is 3.8–4.5, in sexual intercourse the ejaculation of semen normal pH 7.1–7.8) raises the overall pH towards neutral. Nanobodies readily penetrate mucosal surfaces to reach tissues [64], so their potential as microbicides merits further exploration. As discussed in the previous section, functional nanobodies can be readily expressed and secreted by *lactobacilli* including strains that live commensally in the human vagina.

In a preclinical trial on rhesus macaques, R. Le Grand and colleagues used a chimeric simian immunodeficiency virus (SHIV 162P3) bearing an HIV *env* gene in tests of candidate microbicides [65]. Administration of nanobody J3 formulated in hydroxethylcellulose gel 1 hour before a high dose vaginal challenge with SHIV 162P3 protected 8/8 animals from infection. This small preclinical trial may encourage further testing leading to a human trial, possibly with a mixture of broadly neutralizing bi-specific nanobodies that raise the coverage from 96–100% of HIV-1 strains and increase potency [33].

### 3.4. Nanobodies as Labeling Agents to Detect Latent Reservoirs of HIV Infected Cells

Due to their small size, nanobodies are rapidly removed via the kidney from the human body, which is clearly beneficial for imaging in patients. For in vivo imaging, the nanobody should carry a label, but the random labelling of lysine residues often employed frequently results in a substantial reduction of the functionality of the nanobody [66]. Because a nanobody comprises a single amino acid chain, it can be extended at either the N- or the C-terminus with amino acids to which specific fluorescent labels can be attached. Directional labelling via Cys introduced near the C-terminus does not impair specific binding to the antigen, in contrast to random labelling which can be problematic if a basic amino acid residue is present in the CDRs. The use of nanobodies for imaging is more advanced in cancer applications than in virology. For instance, labelling anti-HER2 VHH showed more rapid accumulation in breast tumors than the labelled human mAb trastuzumab (Herceptin), thus providing more specific localization and less background [65]. C-terminal extended VHH to cancer markers is proving to be useful for imaging various carcinomas by computerized tomography, positron emission tomography, and magnetic resonance imaging. Therefore, HIV-1-infected tissues expressing HIV-1 antigens should also be detectable and labeled anti-gp140 VHH may enable the detection of HIV reservoirs in vivo where occasional cells express envelope glycoprotein. As a general tool in cell biology, Pleiner et al. [67] developed specific C-terminal labelling of nanobodies for super-resolution imaging, while Prole and Taylor [7] recently illustrated the use of targeted fluorescent protein tags on nanobodies to visualize and manipulate intracellular signaling pathways in living cells. 

## 4. Conclusions and Prospect

The versatility of nanobodies lends them to several applications in control of human pathogenic viruses [12,13,68], alongside other single chain or mini-antibody constructs [69]. In HIV studies, they show particular promise as diagnostic and imaging agents, in vaccine design, and as microbicides. Neutralizing nanobodies have been useful in defining vulnerable neutralization sites on the HIV-1 envelope. Bivalent nanobodies binding to two separate sites on gp120 and gp41 show up to a 1400-fold increase in potency compared to either nanobody alone [33]. In addition to the expression of nanobodies in commensal lactic acid bacteria for vaginal delivery, the relatively small size of nanobodies and humanized nanobodies indicates that several VHH can be easily incorporated into adenovirus-associated virus or other viral vectors as well as bacteria [56,70] for in vivo control of infection. A recently published mouse model illustrates the potential use in vivo of a chronically secreted nanobody from bacteria binding to CD47, which is an anti-phagocytic receptor often over-expressed in human cancer [70]. Delivery of the anti-CD47 nanobody by tumor-colonizing bacteria increased activation of tumor-infiltrating T cells, resulting in tumor regression and suppression of metastases.

It remains to be determined by direct comparison whether nanobodies have advantages over whole human monoclonal antibodies. For in vivo administration, the longer half-life and lower immunogenicity may favor human mAbs, although this advantage is partially mitigated by use of ‘humanized’ nanobodies; for administration as a topical microbicide, nanobodies may have the edge with their economy of industrial manufacture and formulation, heat and pH stability, penetration of mucosal surfaces, and high expression by commensal vectors. As with anti-tumor VHH, nanobodies against HIV-1 are at the preclinical stage, but they have similar prospects for application to immunotherapy and prevention.

## Figures and Tables

**Table 1:** Nanobodies with broad neutralizing activity for HIV-1 ^a^.

Name of VHH	Domain Recognized ^b^	Breadth of Strains Tested ^c^	Immunogen	Reference
A12	CD4bs	45%	gp120	[25]
C8	CD4bs	45%	gp120	[25]
D7	CD4bs	40%	gp120	[25]
1B5	CoRbs	75%	gp140	[26,33]
1F10	V3 loop	73%	gp140	[26,33]
2E7	gp41 heptad	80%	gp140	[26,33]
J3	CD4bs	96%	gp140	[27,31]
2H10	MPER	45%	gp41-liposomes	[28]
JM2	CD4bs	60%	gp140	[29,30]
JM3	CoRbs	73%	gp140/CD4 mimic	[29,30]
JM4	CD4bs/CoRBs	70%	gp140/CD4 mimic	[29,30]
3E3	CD4bs	95%	gp140	[31,33]
A14	CD4bs	74%	gp140	[31]
B9	CD4bs	77%	gp140	[31]
B21	CD4bs	72%	gp140	[31]
VHH-9	CD4bs	53%	gp140 SOSIP	[32]
VHH-28	CD4bs	65%	gp140 SOSIP	[32]
VHH-A6	CD4bs	77%	gp140 SOSIP	[32]

^a^ Variable domains of heavy-chain only antibodies (VHH) are listed by chronology of their initial publication; each study used llamas (*Lama glama*) for immunization except for Koch et al. [32] who used dromedaries (*Camelus dromedarius*); ^b^ CD4bs—CD4 binding site; CoRbs—coreceptor binging site/CD4 induced domain; MPER—membrane proximate external region of gp41; V3 loop—crown of V3 cryptic site on gp120; gp41 heptad—1st heptad repeat of gp41; ^c^ Proportion of HIV-1 strains neutralized; different studies used different panels of tier 1, tier 2 and tier 3 pseudoviruses; values are approximate for some studies owing to lower number of strains tested; SOSIP—disulfide bond between gp120 and gp41.
